# A novel strategy for the identification of antigens that are recognised by bovine MHC class I restricted cytotoxic T cells in a protozoan infection using reverse vaccinology

**DOI:** 10.1186/1745-7580-3-2

**Published:** 2007-02-09

**Authors:** Simon P Graham, Yoshikazu Honda, Roger Pellé, Duncan M Mwangi, E Jane Glew, Etienne P de Villiers, Trushar Shah, Richard Bishop, Pierre van der Bruggen, Vishvanath Nene, Evans LN Taracha

**Affiliations:** 1 International Livestock Research Institute, P. O. Box 30709, Nairobi 00100, Kenya; 2 The Institute for Genomic Research, 9712 Medical Center Drive, Rockville, MD 20850, USA; 3 Ludwig Institute for Cancer Research – Brussels branch, Avenue Hippocrate 74 – UCL 7459, B-1200 Brussels, Belgium

## Abstract

**Background:**

Immunity against the bovine protozoan parasite *Theileria parva *has previously been shown to be mediated through lysis of parasite-infected cells by MHC class I restricted CD8^+ ^cytotoxic T lymphocytes. It is hypothesized that identification of CTL target schizont antigens will aid the development of a sub-unit vaccine. We exploited the availability of the complete genome sequence data and bioinformatics tools to identify genes encoding secreted or membrane anchored proteins that may be processed and presented by the MHC class I molecules of infected cells to CTL.

**Results:**

Of the 986 predicted open reading frames (ORFs) encoded by chromosome 1 of the *T. parva *genome, 55 were selected based on the presence of a signal peptide and/or a transmembrane helix domain. Thirty six selected ORFs were successfully cloned into a eukaryotic expression vector, transiently transfected into immortalized bovine skin fibroblasts and screened *in vitro *using *T. parva*-specific CTL. Recognition of gene products by CTL was assessed using an IFN-γ ELISpot assay. A 525 base pair ORF encoding a 174 amino acid protein, designated Tp2, was identified by *T. parva*-specific CTL from 4 animals. These CTL recognized and lysed Tp2 transfected skin fibroblasts and recognized 4 distinct epitopes. Significantly, Tp2 specific CD8^+ ^T cell responses were observed during the protective immune response against sporozoite challenge.

**Conclusion:**

The identification of an antigen containing multiple CTL epitopes and its apparent immunodominance during a protective anti-parasite response makes Tp2 an attractive candidate for evaluation of its vaccine potential.

## Background

*Theileria parva *is a tick-transmitted haemoprotozoan parasite that causes an acute and often fatal disease of cattle termed East Coast fever (ECF). ECF continues to be a major threat to smallholder farmers in eastern, central and southern Africa. There are at least 28 million cattle at risk, over one million animals die each year, and annual economic losses are estimated to be US$ 189 million [1]. The multinucleate schizont stage of the parasite has the remarkable ability to transform the host lymphocytes it infects into a state of uncontrolled proliferation [2]. Whilst this allows the rapid propagation of the parasite it also leads to pathology and ultimately death in susceptible animals [3]. Cattle can be immunized against infection by the simultaneous infection of animals using the tick-derived infective sporozoite stage of the parasite and treatment with long-acting tetracycline [4]. This infection and treatment regime engenders a robust and long-lasting protective immunity that is mediated by MHC class I restricted CD8^+ ^cytotoxic T lymphocytes (CTL) directed against the schizont-infected lymphocyte [5-7].

The post-genomics era offers an opportunity to develop new strategies for vaccine development. In particular, there has been a paradigm shift in the approach to antigen discovery as highlighted by the use of the complete genome sequence of *Neisseria meningitidis *to identify and test a list of candidate vaccine antigens against meningococcal meningitis [8]. The success of this approach, termed 'reverse vaccinology' coupled with the ever-growing number of bacterial and protozoan genomes being sequenced has allowed this concept to be extended to other pathogens [9-11]. Successful application of reverse vaccinology is dependent upon the availability of a high-throughput system to test candidate antigens, but it has been hampered by the lack of good *in vitro *correlates of immunity. Protection from disease upon challenge in small animal models is normally the only read-out available [12]. The correlation between the induction of CTL responses and immunity to *T. parva *offered a system with which to apply reverse vaccinology to a complex pathogen where immunity was cell-mediated.

The strategy involved analyzing the genome to identify genes encoding secreted or membrane bound proteins. Since the genes were expressed by the intra-lymphocytic schizont stage these proteins would be exposed to the host cell cytosol and hence available for presentation to CD8^+ ^CTL through the MHC class I processing and presentation pathway. Selected genes would be screened *in vitro *for recognition by *T. parva *specific CTL. This report describes the successful application of this novel approach to identify a vaccine candidate antigen of *T. parva *that is the target of schizont-specific CTL from immune cattle.

## Results

### Screening of genes selected from genome data

**Figure 1:**
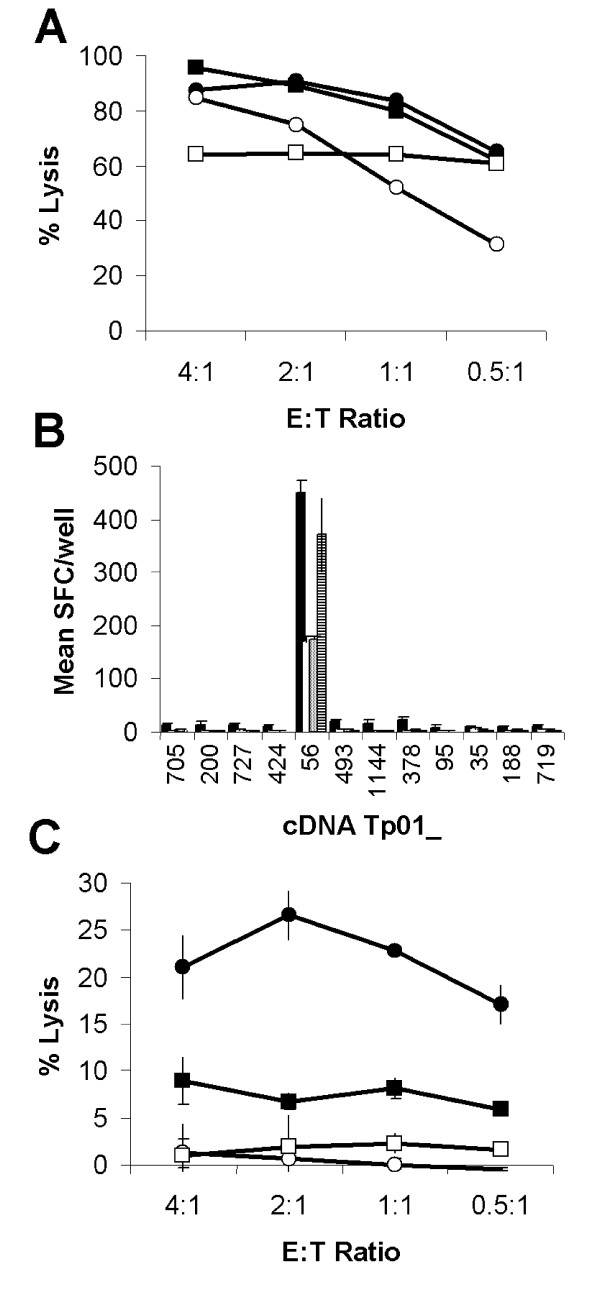
**Identification of antigen Tp2**. (**A**) Lysis of autologous schizont infected cells by four *T. parva*-specific CTL lines capable of (**B**) specifically reacting to iSF transfected with 12 selected genes as assessed by IFN-γ ELISpot and (**C**) lysis of cDNA Tp01_56 transfected-iSF. (**A**) Closed circles: BW002; open circles: BW013; closed squares: BW014; and open squares: D409. (**B**) IFN-γ responses to selected gene transfected iSF are presented as mean numbers of spot forming cells (SFC)/well. Black: BW002; white: BW013; grey: BW014; and hatched: D409. (**C**) Mean lysis of cDNA Tp01_56 transfected-iSF by CTL from the four cattle. Closed circles: cDNA Tp01_56 transfected autologous iSF; open circles: Irrelevant gene transfected autologous iSF; closed squares: cDNA Tp01_56 transfected autologous iSF pre-incubated with an anti-MHC class I mAb; and open squares: cDNA Tp01_56 transfected allogeneic iSF.

The preliminary sequence data of *T. parva *(Muguga) chromosome 1 [13] was run through the gene finding programs GlimmerM [14] and Phat [15], and the proteins encoded by the predicted *T. parva *genes subjected to a variety of analyses to identify secreted or membrane-anchored proteins. Fifty-five ORFs encoding candidate antigens were selected for screening, of which 36 were successfully cloned into pTargeT [16]. Each of the 36 ORFs were transiently transfected into autologous immortalized skin fibroblasts (iSF) and screened with a panel of schizont specific polyclonal CTL lines or CTL clones generated from 13 *T. parva *immune cattle representing a spectrum of bovine leukocyte antigen (BoLA) class I genotypes. CTL from four animals (BW002, BW013, BW014 and D409) exhibiting high lytic activity against autologous schizont-infected lymphoblasts (Fig. 1A), all showed significant IFN-γ ELISpot responses to iSF transfected with cDNA Tp01_56 (Fig. 1B; p < 0.01). CTL lines from the other nine cattle did not recognise iSF transfected with any of the selected ORF's and no false positives were detected during the screening (data not shown). The ability of CTL to lyse iSF transfected with cDNA Tp01_56 was assessed using a ^51^Chromium release assay (Fig. 1C). CTL exhibited specific lysis against cDNA Tp01_56 transfected cells and not against cells transfected with an irrelevant cDNA cloned into the same vector. Lysis of cDNA Tp01_56 transfected cells by CTL was inhibited by pre-incubation of target cells with a blocking mAb against MHC class I (IL-A88; ILRI, Nairobi, Kenya). This observation together with the inability of CTL to lyse cDNA Tp01_56 transfected allogeneic cells suggested that the recognition and lysis was MHC class I restricted. The gene encoding cDNA Tp01_56 was assigned the name Tp2 (GenBank accession number XM_760490).

### Molecular characterisation of *T. parva *CTL target antigen Tp2

**Figure 2:**
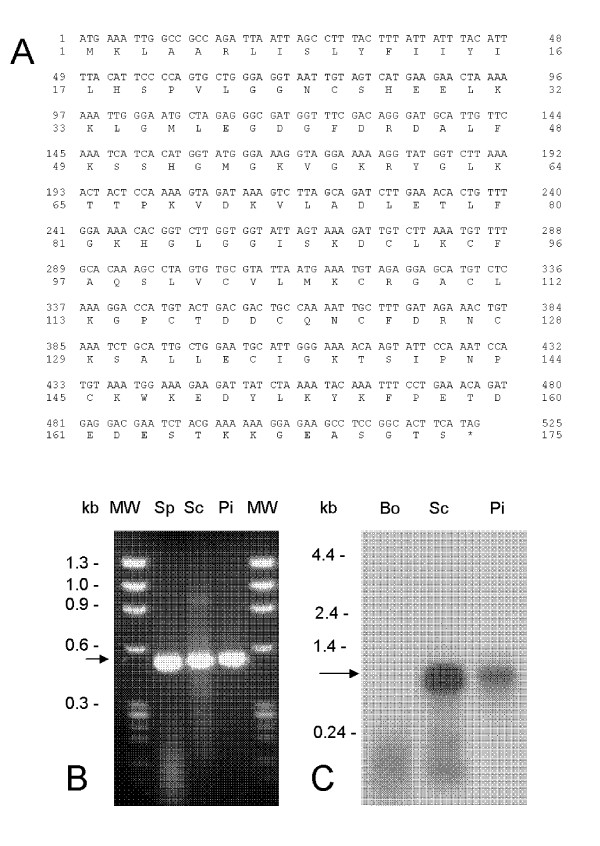
**Nucleotide and deduced amino acid sequence of antigen Tp2 from *T. parva *Muguga**. (**A**) Tp2 is encoded by a single ORF of 525 bases translating into a protein of 174 amino acids. Transcription was assessed by (**B**) RT-PCR from RNA isolated from sporozoite (Sp), schizont (Sc) and piroplasm (Pi) developmental stages of *T. parva *and by (**C**) Northern blot hybridization against bovine (Bo), schizont (Sc) and piroplasm (Pi) RNA.

The Tp2 gene encodes 174 amino acid residues, has a calculated mass of 19,141Da and an N-terminal signal peptide with a predicted cleavage site between residues 23 and 24. The predicted mature protein contains twelve cysteine amino acid residues suggesting that the tertiary structure may be dependent on di-sulfide linkages. The nucleotide and deduced amino acid sequences of the Tp2 gene are shown in Fig. 2A. Reverse transcriptase(RT)-PCR revealed transcription of Tp2 in sporozoite, schizont and piroplasm life-cycle stages of *T. parva *(Fig. 2B) and Northern blots detected the presence of ~1 kb transcript in schizont and piroplasm RNA (Fig. 2C). Transcriptome analysis of *T. parva *using massively parallel signature sequencing indicated that Tp2 is expressed at a relatively high level (1069 tpm) in the schizont stage [17]. The cDNA clone was isolated from the schizont cDNA library contains a 1026 bp insert with 105 and 396 bp 5' and 3' UTR's, respectively. Searches of protein and DNA databases using BLAST identified a homolog of Tp2 in *Theileria annulata*, a molecule described as a membrane-associated antigen, TaD [18].

### Mapping of CTL epitopes on Tp2

**Figure 3:**
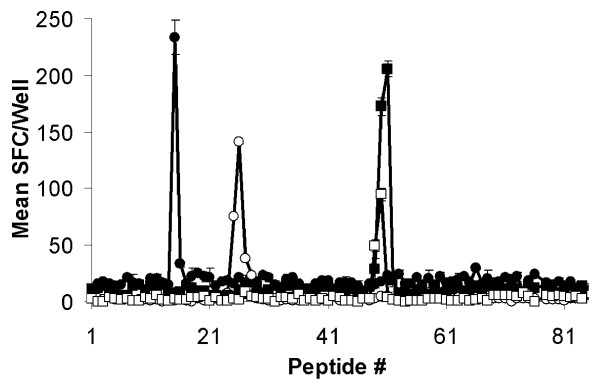
**Mapping of CTL epitopes on Tp2**. Eighty-four 12-mer peptides overlapping by ten amino acids encompassing the full length of Tp2 were screened with four CTL and recognition assessed by IFN-γ ELISpot. Responses are presented as mean numbers of spot-forming cells (SFC)/well. Closed circles: BW002; open circles: BW013; closed squares: BW014; and open squares: D409.

The CTL epitopes on Tp2 were mapped using a library of 12-mer overlapping synthetic peptides covering the full length of the Tp2 protein. Recognition of Tp2 peptides was assessed by IFN-γ ELISpot using autologous iSF as antigen-presenting cells. Fig. 3A shows the results of screening the Tp2 peptide library with CD8^+ ^polyclonal CTL lines from animal BW002, BW013, BW014 and CTL clone T29.10 from animal D409. This allowed the identification of four epitope-containing regions. Minimal length antigenic peptides were defined by screening all possible 8-, 9-, 10- and 11-mers covering the regions identified above. The four minimal length antigenic peptides recognized by CTL were 11-mers, SHEELKKLGML (BW002) and KSSHGMGKVGK (BW013) and overlapping 9-mers, FAQSLVCVL (D409) and QSLVCVLMK (BW014) (data not shown).

### Tp2 specific responses during protective immunity

**Figure 4:**
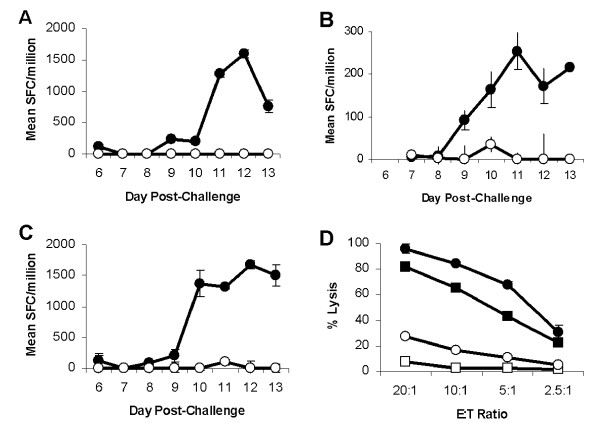
***Ex vivo *Tp2 specific CD8+ T cell responses following challenge of immune cattle**. Tp2 specific CD8^+ ^T cell responses following challenge of *T. parva *immune cattle. Responses of three cattle, (**A**) BW014, (**B**) BW013 & (**C**) BW002, were measured longitudinally by IFN-γ ELISpot and responses are presented as mean numbers of spot-forming cells (SFC)/1 × 10^6 ^CD8^+ ^T cells. Closed circles: Tp2 antigenic peptide and open circles: control peptide. (**D**) Cytotoxic activity of stimulated BW002 PBMC was assessed 14 days post-challenge. Closed circles: Autologous schizont-infected cells; closed squares: Tp2 antigenic peptide pulsed iSF; open squares: Control peptide pulsed iSF; and open circles: Allogeneic schizont infected cells.

As a prelude to directly testing the vaccine potential of the Tp2 antigen in cattle, an experiment was conducted to look for a Tp2-specific component within the protective CD8^+ ^T cell response following the challenge of three ECF immune cattle with a lethal dose of sporozoites. All animals were solidly resistant to challenge, exhibiting only a transiently detectable parasitosis and no fever (data not shown). CD8^+ ^T cells enriched from peripheral blood responded to Tp2 antigenic peptides as measured by IFN-γ ELISpot assay (Fig 4). Responses to the Tp2 antigenic peptides were significantly greater than those to peptides from other regions of Tp2 from day 9–11 post-challenge (Fig. 4A–C; p < 0.05). Both the kinetics and magnitude of the response correlate well with those previously described for schizont-specific CTL in efferent lymph and blood following challenge of immune animals [5]. Attempts to detect schizont and Tp2 specific cytotoxic activity directly in the blood of cattle post-challenge failed to demonstrate any significant lysis (data not shown). However, 14 days post-challenge, BW002 PBMC stimulated *in vitro *with autologous irradiated schizont- infected cells and tested for cytotoxicity 7 days later exhibited significant cytolytic activity against both schizont-infected cells and iSF pulsed with the Tp2 antigenic peptide SHEELKKLGML (Fig. 4D; p < 0.001). This finding is consistent with the hypothesis that the CTL precursor frequency in peripheral blood was too low to be measured directly but could be increased to detectable levels following a single re-stimulation *in vitro*.

## Discussion

From the 986 predicted ORFs on chromosome 1, bioinformatics was used to select 55 genes encoding proteins that could potentially access the host cell cytosol. From the selected genes, 36 were successfully cloned into a eukaryotic expression vector and screened for recognition by schizont-specific CD8^+ ^CTL derived from 13 cattle immunised against *T. parva *by a live infection and treatment immunization regime. One antigen, termed Tp2, was identified by CTL from 4 different animals. Subsequent analysis has defined multiple CTL epitopes on this molecule. Further evidence of the status of Tp2 as a bovine CTL target antigen was provided by the temporal correlation of detectable Tp2 specific CD8^+ ^T cell responses with the onset and clearance of *T. parva *schizont parasitosis following challenge infection.

The success of this approach has been underpinned by the development of a sensitive CTL screening assay with which to detect recognition of the selected antigens expressed by transiently transfected antigen-presenting cells. The current screening system is based on the same principle as an assay developed to identify target antigens of melanoma tumor cell specific human CTL [19,20]. This system utilized the WEHI TNF-α bioassay to detect CTL recognition of COS-7 cells co-transfected with pools of melanoma cell cDNA and appropriate HLA class I cDNA [19,20]. The limited number of cloned full length BoLA class I cDNA and the relative insensitivity of WEHI cells to bovine TNF-α meant that an alternative antigen-presenting cell and read-out system were required to screen for *T. parva *antigens with bovine CTL. Autologous bovine skin fibroblasts stably transfected with the large T antigen of SV40 provided a system that did not limit the BoLA haplotypes that could be recruited for screening, avoided the practical limitations of working with primary cell lines and gave significantly enhanced expression of proteins following transient transfection. All bovine schizont-specific CTL lines tested to date secrete IFN-γ and detection of this cytokine using an ELISpot assay [21] provided an extremely sensitive read-out to replace the TNF bioassay. Screening of the selected genes with schizont-specific CTL identified cDNA Tp01_56 as encoding a target antigen. We subsequently confirmed that recognition was MHC class I restricted and led to lysis of the expressing cell. Despite the relative inefficiency of transient transfection, ^51^Chromium release assays could be performed to confirm MHC class I restricted lysis of Tp2 transfected cells.

A strength of this approach is the ability to use schizont-specific CTL populations that have been maintained in the laboratory to screen for antigens. However, although CTL lines present a sensitive and exquisitely specific screening tool there is the possibility that the antigens that will be identified will be the most immunodominant as a result of biases introduced during the *in vitro *restimulation procedure that is required to maintain the lines. The antigens identified may represent those that are well expressed in schizont-infected cell lines *in vitro *and may have little relevance to protective CTL responses *in vivo*. As a prelude to testing this hypothesis directly with challenge protection experiments, an experiment was conducted to look at Tp2-specific CD8^+ ^T cell responses following the challenge of immune animals with a lethal dose of sporozoites. Both, the kinetics of the response, observed from day 9 post-challenge, and the magnitude of the response, with a responder frequency of 1/500, correlates very well with that previously described for schizont-specific CTL in efferent lymph and peripheral blood following challenge of immune cattle [5] and provides evidence to support a role for Tp2 in protective immunity. To this end, in a preliminary immunization and challenge cattle experiment evaluating Tp2 and other candidates, it was demonstrated that this CTL target antigen has potential for vaccine exploitation [16].

The ultimate vaccine against *T. parva *will almost certainly need to incorporate multiple antigens and epitopes in order to confer protection in the genetically diverse out-bred cattle population exposed to challenge by antigenically diverse parasite populations in the field. This study demonstrates proof of concept for the application of a genomic approach for identification of candidate antigens for inclusion in vaccines designed to induce CTL-based protection. This approach compliments the random screening of cDNA libraries that has successfully been applied to identify additional CTL target antigens from *T. parva *[16] and human melanomas [19,20]. The major challenge remains the formulation and delivery of these antigens in a manner that adequately primes protective memory CD8^+ ^T cell populations *in vivo*.

## Conclusion

This study validates the exploitation of genomic data to identify vaccine candidate antigens from a complex haemoprotozoan parasite using CTL derived from the natural ruminant host. Extension of this approach to the complete genome of *T. parva *using CTL restricted by additional bovine haplotypes is likely to result in identification of further vaccine candidates. The identification of an antigen containing multiple CTL epitopes makes Tp2 an attractive candidate for inclusion in a CTL targeted sub-unit vaccine and also for the study of polymorphism in CTL epitopes in cattle and Cape buffalo (*Syncerus caffer*). An in-depth evaluation of this antigen in cattle involving a range of antigen delivery technologies perceived to have the potential to induce bovine CTL is in progress in order to assess the immunogenicity and protective efficacy of Tp2 against clinical East Coast fever

## Methods

### Generation of selected gene list

Preliminary contigs of the *T. parva *genome were searched against a non-redundant database of proteins extracted from GenBank. The search results were used to produce a set of putative *T. parva *genes that encoded highly conserved eukaryotic genes. This set of genes was complemented with a number of full length genes isolated from a cDNA library made from purified schizonts and were used to train the gene finding programs GlimmerM [14] and Phat [15], which were subsequently run against all of the preliminary contigs to produce gene models for the entire preliminary genome sequence. The proteins encoded by the predicted *T. parva *genes on chromosome 1 were searched against a non-redundant protein database using WU-BLAST [22], and predictions of signal peptides and signal anchors [23] and transmembrane domains [24] analysed. The results were reviewed and a set of 55 genes encoding candidate antigens was selected for cloning and screening [16].

### Cloning of selected genes

Open reading frames (ORFs) less than 3.1 kb were successfully amplified by OneStep RT-PCR kit (QIAGEN Ltd., Crawley, UK) using RNA purified from *T. parva *(Muguga) schizont-infected lymphoblasts. Thermal cycles were: Genes up to 1 kb: 50°C 30 minutes; 95°C 15 minutes; 94°C 30 seconds; 55°C 30 seconds; 72°C 1 minute; 35 times from 94°C cycle and finally 72°C 10 minutes. Genes between 1 and 2 kb: 50°C 30 minutes; 95°C 15 minutes; 94°C 1 minute; 55°C 1 minute; 72°C 1 minute; 35 times from 94°C cycle and finally 72°C 10 minutes. Genes between 2 and 3 kb: 45°C 30 minutes; 95°C 15 minutes; 94°C 10 seconds; 55°C 1 minute; 68°C 3 minutes; 35 times from 94°C cycle and finally 68°C 10 minutes. Amplified genes were purified from agarose gels by QIAquick Gel Extraction kit (QIAGEN) and cloned into eukaryotic expression T-vector pTargeT (Promega, Madison, WI, USA). Ligated samples were electroporated into JM109, and colonies were screened by PCR using gene specific internal forward primers and vector specific reverse primer (5'GAGCGGATAACATCACACAGG3'). Positive colonies were cultured in 2 ml and plasmids purified by QIAprep Spin Miniprep kit (QIAGEN) and sequenced by ABI PRISM 377 DNA sequencer (Applied Biosystems, Foster City, CA, USA). Although 51 of 55 selected genes were amplified, 36 were cloned successfully in pTargeT [16]. Some of the genes were consistently cloned in wrong orientation suggesting the gene products were toxic to *E. coli*. Some genes were amplified by the reverse primer only and missed the 5' end.

### Analysis of the temporal expression of Tp2

Total RNA from sporozoites, schizonts and piroplasms was reverse-transcribed into ss-cDNA by reverse transcriptase using oligo-(dT) as primer following the recommendation of the supplier (Invitrogen). After removal of RNA complementary to the cDNA by treatment with RNase H and purification with phenol-chloroform, the ss-cDNA was PCR-amplified in the presence of specific primers used to generate the Tp2 ORF (5'-ATGAAATTGGCCGCCAGA-3' and 5'-CTATGAAGTGCCGGAGGCTTCTCC-3'). Northern blots of RNA from the schizont and piroplasm stages of *T. parva *were probed with the Tp2 ORF DNA fragment. This probe was also used to screen a schizont cDNA library and restriction enzyme analysis was performed by digestion with *Eco *RI and *Bam *HI.

### Infection and treatment immunization of cattle and generation of CTL

All animal experimentation was reviewed and approved by the ILRI Institutional Animal Care and Use Committee. Four Boran (*Bos indicus*), 1 Jersey *(Bos taurus)*, 1 Holstein-Friesian *(Bos taurus) *and 4 crossbreds, selected on the basis of BoLA type, were immunized against *T. parva *Muguga stock, challenged after 3 months and schizont-specific polyclonal CTL lines and clones established as described [16].

### Transient transfection of immortalized skin fibroblasts

Immortalized skin fibroblasts (iSF) were transfected in 96-well plates with cDNA clones (100 ng/well) using Fugene-6 (Roche Diagnostics GmbH, Mannheim, Germany) and cultured for 24 hours.

### IFN-γ ELISpot and ^51^Chromium release assays

Transfectants were co-cultured with schizont-specific CTL and recognition assessed using an IFN-γ ELISpot assay [16]. Transfectants and schizont-infected cells were labelled with ^51^Chromium (Amersham Biosciences Europe GmbH, Freiburg, Germany) and lysis by schizont-specific CTL lines assessed [25].

### Identification of CTL epitopes with synthetic peptides

A peptide library of Tp2 was generated that contained every 12 mer, 11 mer, 10 mer and 9 mer offset by 2 amino acids from the protein sequence (Cleaved PepSet; Mimotopes, Clayton, Australia). Peptides were prepared by truncations of 12 mers at the N-terminus and supplied lyophilized with each tube containing a nominal 12 mer and the 9, 10, 11 mer truncations with the same C-terminus. Peptides were dissolved in 400 μl 50% (v/v) DNA synthesis grade acetonitrile/water (Applied Biosystems). Peptides were further diluted to 10 μg/ml in complete RPMI-1640 and 10 μl added to triplicate wells of an ELISpot plate. Autologous iSF were adjusted to a density of 4 × 10^5^/ml and 50 μl added to wells containing peptides. The plates were incubated at 37°C for 1 hour before 50 μl of CTL at a density of 2 × 10^5^/ml were added to each well. Plates were incubated and developed as described above. Based on the results of screening the peptide library, individual 8, 9, 10 or 11 mer peptides were synthesized and tested as described above. Peptide-pulsed iSF were prepared as targets for ^51^Chromium release assays by overnight incubation with peptide at 1 μg/ml in complete DMEM. Cells were harvested, labelled and assayed as described above.

### *Ex vivo *detection of Tp2 specific CD8^+ ^T cell responses

Immune cattle, BW002, BW013 and BW014, whose schizont-specific CTL had recognized Tp2, were challenged with a lethal dose of *T. parva *(Muguga) sporozoites and bled daily from day 6 to 13 post-challenge. CD8^+ ^T cells and CD14^+ ^monocytes were purified from PBMC by MACS magnetic cell sorting according to the manufacturer's instructions (Miltenyi Biotec, Gergisch Gladbach, Germany). CD8^+ ^T cells were sorted indirectly using a monoclonal antibody specific for bovine CD8 (IL-A105; ILRI, Nairobi, Kenya) followed by incubation with goat anti-mouse IgG microbeads (Miltenyi Biotec). CD14^+ ^monocytes were sorted directly by incubation with CD14 microbeads (Miltenyi Biotec). CD8^+ ^T cells (2.5 × 10^5^/well) and CD14^+ ^monocytes (2.5 × 10^4^/well) were added to IFN-γ ELISpot plates containing Tp2 peptides (1 μg/ml final concentration) and were incubated and developed as described [21]. To recall Tp2 specific CTL responses, PBMC were stimulated with autologous infected lymphoblasts 14 days post-challenge, viable cells were harvested 7 days post-stimulation and lytic activity against infected lymphoblasts and Tp2 peptide pulsed iSF assessed as described above.

### Statistical analysis

Analysis of variance (ANOVA) was used for the analysis of fixed effects on different traits using SAS Release 8.2 (SAS Institute Inc., Cary, USA).

## Competing interests

The author(s) declare that they have no competing interests.

## Authors' contributions

SG carried out the immunological assays and drafted the manuscript. EPdV, TS and RB annotated and analyzed the genome data. YH cloned the selected genes. RP conducted the molecular analysis of Tp2. DM and EJG conducted some of the immunological assays. PvdB participated in the design of the study. VN and ELNT conceived the study and participated in the design and coordination, and helped draft the manuscript. All authors have read and approved the final manuscript.
